# The Perfect Predator: A Scientist’s Race to Save Her Husband, by Drs. Steffanie Strathdee and Thomas Patterson

**DOI:** 10.5334/aogh.2997

**Published:** 2020-08-07

**Authors:** Pia D. M. MacDonald

**Affiliations:** 1 RTI International, Research Triangle Park, NC, US; 2 Department of Epidemiology, Gillings School of Global Public Health, University of North Carolina, Chapel Hill, NC, US

## Abstract

This is a book review of “The Perfect Predator: A Scientist’s Race to Save Her Husband” by Drs. Steffanie Strathdee and Thomas Patterson.

Reading this book as the COVID-19 pandemic was emerging had a very sobering impact on me. It was harrowing to read it the week my septuagenarian mother boarded a cruise ship on the Nile, while another ship was disembarking with many COVID-19 cases. The author, Dr. Patterson, acquired an infectious disease in Egypt while cruising on the Nile and was “treated” there while simultaneously acquiring a multidrug resistant bacterial pathogen at a hospital. He almost lost his life.

It was bold of Drs. Steffanie Strathdee and Thomas Patterson to publish their Kafkaesque and highly personal medical journey. The reader gets an in-depth look at them as people, scientists, parents, mentors, teachers, colleagues, and friends. This book weaves several stories into a tapestry of medical drama, public health, and personal triumph. One story is a personal account of having a deathly ill spouse, with dwindling treatment options. Another story details a scientist’s determination and race to find a needle-in-a-haystack treatment for her husband, with highlights of the history of epidemiology and medicine (Dr. John Snow – field epidemiology, Dr. Alexander Langmire – discovery of antibiotics), including a notable description of phage therapy first documented in 1896 but with limited use in Western medicine [1]. Ultimately, the account is a critique of how we, as a society, have voyaged all too fast into an age where antibiotics may or may not work to treat a bacterial disease.

Two salient parts of the book caught my attention. First is the compelling first-hand account of the use of bacterial viruses to treat bacterial infections (phage therapy) and how this course of treatment has been adopted by some countries, while being discounted and neglected in others like the United States [1]. Second, the authors shine a spotlight on the global problem of the growing emergence of lethal bacterial pathogens that are resistant to multiple antibiotic treatments, which we know is getting worse each year [2]. They show how easy it is for an American to be directly impacted by a public health problem, such as antibiotic resistance, in another country (Egypt) far from our own. This is not to show that antibiotic resistance is a distant health security threat, but to show the common vulnerabilities of our global public health systems.

The book begins with some background about the authors and their lives as scientists who work around the world. Next, they describe a vacation trip to Egypt and cruise on the Nile, and this is where the medical journey begins with a gastrointestinal illness and visit to an Egyptian medical clinic. From there, we follow them from Egypt on a medical evacuation to Frankfurt, Germany, and another flight to the university-associated hospital at the University of California at San Diego. The chronological structure of the book at times got monotonous and detailed, with the day-to-day medical care in an extraordinarily long nine-month hospital stay in San Diego. That magnification may distract a reader from some of the other fascinating facets the authors bring to light. I found the last resort global quest for an alternative treatment strategy very interesting and well described.

Drs. Strathdee and Patterson begin the book with an Albert Camus quote from *The Plague*, “There have been as many plagues as wars in history; yet always plagues and wars take people equally by surprise.” While the 2019 novel coronavirus (SARS-CoV-2) pandemic did surprise me (I instead expected a highly pathogenic avian influenza pandemic), we cannot hide behind a veneer of surprise for the significant emergence of multidrug resistant bacteria for which we do not have adequate treatments. For almost as long as we have had antibiotics, we have known about resistance and how it occurs, yet we have not done enough to mitigate this impending pandemic.

Another prescient quote rings ever true today in the midst of a global pandemic of a lethal novel virus, “Isolationism is no friend of true science.” With COVID-19 and with the growing number of pathogens and cases of disease with drug-resistant bacteria, we must tackle this health threat now as a global community. We are only as safe as our least safe country, so if drug-resistant bacteria live in one location on the globe, it’s a threat to us all, just like we have seen of SARS-CoV-2. This book is an excellent read for those with interest in global health, antimicrobial resistance, and phage therapy. It adds to the growing collection of accessible books that explain public health threats, past, present, and future to broad audiences.
